# Pulmonary infection by *Mycobacterium riyadhense*: first case report in United Arab Emirates

**DOI:** 10.3389/fmed.2024.1399381

**Published:** 2024-08-21

**Authors:** Batool A. Sawan, Leen O. Saleh, Dina Z. Al Shaltouni, Mohammad A. Sawan, Shefa Gawish, Samar Ahmed, Julio Gomez-Seco, Michael E. Otim

**Affiliations:** ^1^Department of Public Health, Dubai Medical College, Dubai, United Arab Emirates; ^2^Department of Medicine, Near East University, Nicosia, Cyprus; ^3^Fakeeh University Hospital, Dubai, United Arab Emirates

**Keywords:** non-tuberculous mycobacteria, *Mycobacterium riyadhense*, pulmonary cavity, chronic cough, antitubercular drugs

## Abstract

*Mycobacterium riyadhense* is an emerging slowly growing species that belongs to the group of nontuberculous mycobacteria (NTM) with approximately 20 cases reported worldwide. We highlight the first case of pulmonary infection by *Mycobacterium riyadhense* in United Arab Emirates (UAE). A 44-year-old female presented with chronic productive cough; a bronchial breathing pattern was appreciated on auscultation of her right upper lung. She was treated multiple times with allergic medications and antibiotics. Thorough investigations revealed *Mycobacterium riyadhense* and antitubercular drugs were started, eventually she was cured, however she had multiple relapses later. This case report holds a significant potential to make considerable contribution to the diagnosis of NTM, primarily because it presents the first documented case in UAE, as well as insights on how to address possible similar cases in the future.

## Introduction

*Mycobacterium riyadhense* (*M. riyadhense*), is a newly recognized slow-growing, non-photochromogenic NTM. *Mycobacterium riyadhense* was first isolated from a patient in Riyadh, Kingdom of Saudi Arabia (KSA) who presented with maxillary sinus infection in 2009 (1). To-date, about 20 cases have been reported in the literature.

*Mycobacterium riyadhense* seems to be capable of causing a spectrum of clinical presentations that are clinically indistinguishable from Tuberculosis (TB) (2). Despite pulmonary involvement being the most common form of non-tuberculous mycobacterial infections, a range of extra-pulmonary presentations have been reported. The clinical and radiologic findings of pulmonary infection caused by *M. riyadhense* are indifferent from those caused by *M. tuberculosis*, which is the most important human pathogen of the *Mycobacterium tuberculosis* complex (MTBC) (3).

## Case presentation

A previously healthy 44-year-old female of German origin, living in UAE for more than 8 years, was referred to our care by an internist. She presented at Fakeeh University Hospital in Dubai, United Arab Emirates, in November 2021 with a chronic cough and purulent sputum lasting for 1 year. Earlier in mid-2020, she developed a persistent dry cough and sought medical attention from a general practitioner. Following an allergy test that revealed her sensitivity to pollen, she was prescribed Antihistamines, which were ineffective in alleviating her symptoms.

In May 2021, she was inaccurately treated in a local clinic for a bacterial infection with Ampicillin, which unfortunately triggered an allergic reaction. In September 2021, she returned to the local clinic with new onset yellowish sputum and was prescribed another course of antibiotics that the patient does not recall. In November 2021, she experienced a recurrence of the same symptoms, which drove her to Fakeeh University Hospital for medical advice. However, she did not exhibit any significant weight loss, night sweats, fever, or hemoptysis. Her past medical and family history is insignificant. She is a nonsmoker, a married housewife who lives at home with her family, none of whom are experiencing similar symptoms. Additionally, she stated having not had contact with TB patients and stated that there are no pets at home. It is also worth mentioning that she has never received the Bacille Calmette-Guerin (BCG) vaccine and travels annually to her homeland during the holidays.

**Figure 1:**
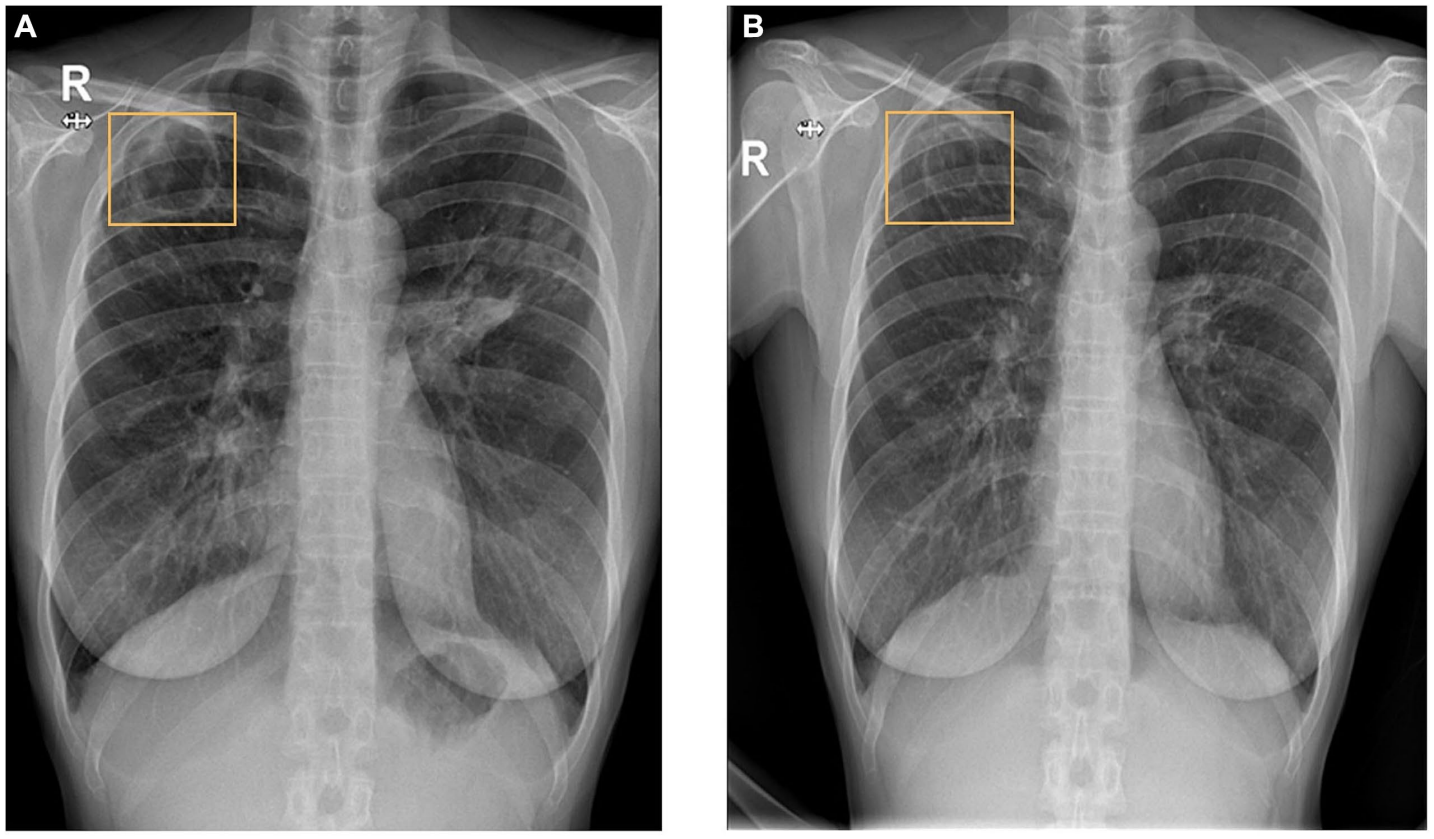
A well-defined cavity in the right upper lung measuring 4.5 cm and patchy consolidation in the left upper and middle lobes in November 2021 **(A)**. Over time, the cavity reduced in size to 3.5 cm then to 3.2 cm with regression of the infiltrates in the left upper lobe in December 2022 **(B)**.

Generally, she appeared alert, oriented, well-nourished, and not in distress. The physical examination showed no cervical lymphadenopathy. Auscultation of the chest revealed normal bilateral vesicular breathing, with a bronchial breathing pattern noted in the right upper lung. Sputum samples were collected for further investigations. In addition, a chest *x*-ray was done, revealing a pulmonary cavity as shown in Figure 1. The sputum culture, conducted over a 6-week period, reported growth of *Mycobacterium riyadhense* in December 2021 as seen in Table 1, with drug sensitivity tested and showed susceptibility to all first- and second-line anti-TB medications. Consequently, a 6-month course of anti-TB medications was initiated, consisting of 2 months of Isoniazid, Rifampin, Pyrazinamide, and Ethambutol, followed by 4 months of Isoniazid and Rifampin. Additionally, vitamin B6 supplements were given, along with a battery of serial investigations to detect any possible medication side effects. These side effects include: visual field assessment; uric acid levels; and liver function tests were monitored monthly and were all nearly normal. After completing the anti-TB regimen, symptoms subsided with radiological images showing improvement.

**Table 1:** Timeline of disease course and serial investigations.

Date	Symptoms	Investigation/Intervention	Result
Nov./2021 (outpatient)	Chronic productive cough with yellowish sputum for 3 months.	Sputum AFB stain	After 2 days came Positive +3.
		PCR	After 1 week came positive NTM.
		LJ culture for 6 weeks	After 6 weeks came positive (*Mycobacterium Riyadhense*).
Feb./2022 (during first anti-TB course)	Asymptomatic	AFB stain for sputum	Negative
		LJ medium for culture	Negative
July/2022 (first relapse) (outpatient)	Fever and cough	PCR	After 1 week came positive NTM.
Aug./2022	Asymptomatic	CTD panel	All negative
		IgA, IgM, IgG, and A1AT	Normal
		HIV IgM and IgG	Negative
Sept./2022 (outpatient)		Bronchoscopy with biopsies	Trachea showed diffuse inflammation. Right bronchial tree: inflammation with whitish phlegm and transbronchial biopsy taken from the posterior segment of RUL showed Necrotizing granuloma in submucosa. Left bronchial tree: inflammation with whitish phlegm with stenosis of 60% in the anterior segment of LUL and the biopsy taken at the entry of it was benign. Bronchoalveolar lavage done and when tested was acellular, Micro negative, AFB negative, and cytology negative.
Jan./2023 (second relapse) (outpatient)	Fever, cough, night sweats, and chest pain	CRP	CRP 11 mg/dL
		ESR	ESR 28 mm/h
		Sputum AFB	AFB Positive +4
Feb./2024 (inpatient)	Cough	Right upper lobectomy (admitted for 5 days)	Specimen showed caseating granulomas with no evidence of malignancy or vasculitis.
	1 week after surgery: Fever, productive cough, and night sweats.	Thoracocentesis (admitted for 4 days)	Pleural fluid analysis: 98% lymphocytes consistent with mycobacterial infection.

AFB, Acid fast Bacilli; PCR, Polymerase chain reaction; LJ, Lowenstein–Jensen; CTD, Connective tissue disease; HIV, Human immunodeficiency virus; RUL, Right upper lobe; LUL, Left upper lobe; CRP, C-reactive protein; and ESR, Erythrocyte sedimentation rate.

**Figure 2:**
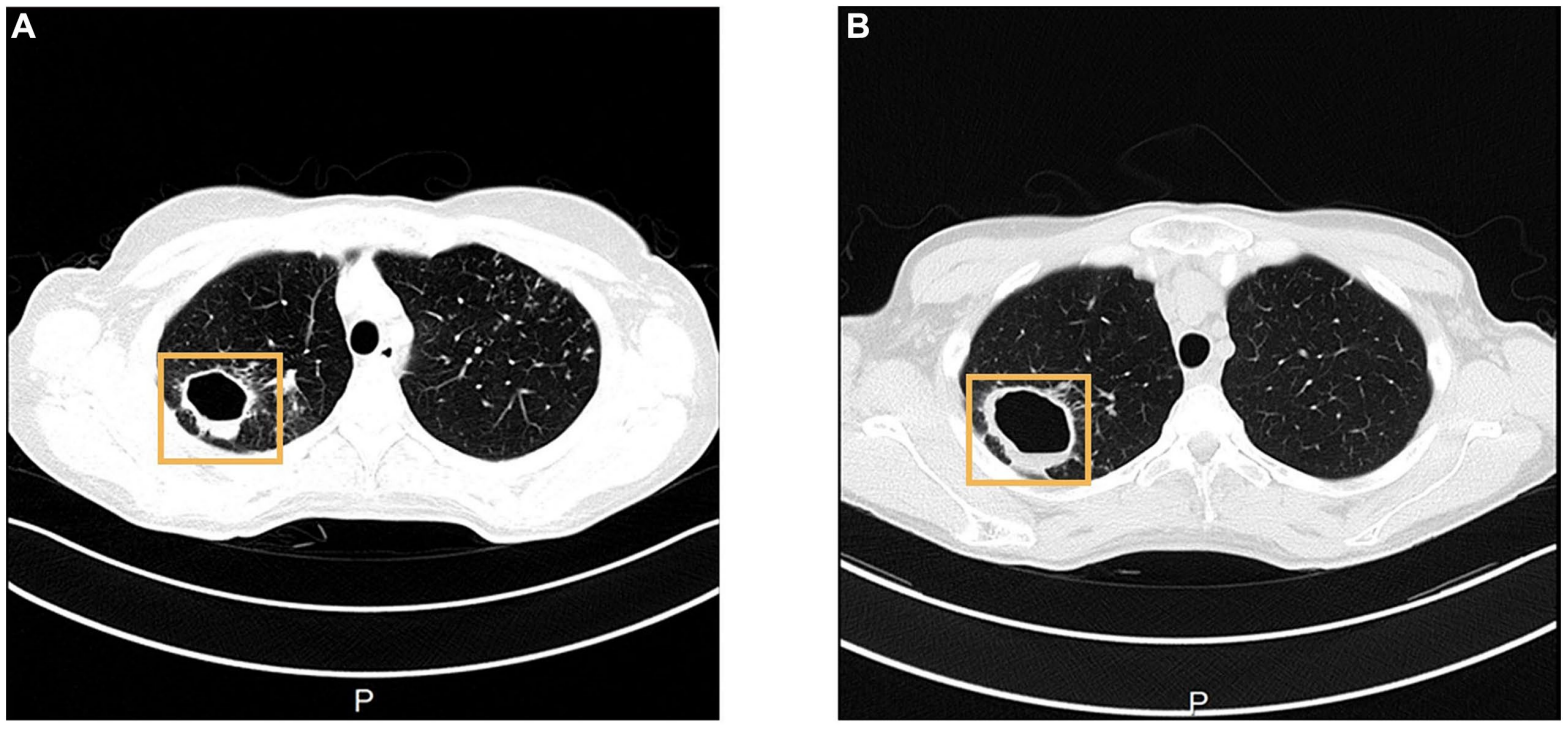
CT shows an increase in the size of right upper lung cavity, from 3.2 cm on 31st August 2022 **(A)**, to 4.5 cm on 16th January 2023 **(B)**. In the CT image, A stands for Anterior, and P stands for Posterior.

**Figure 3:**
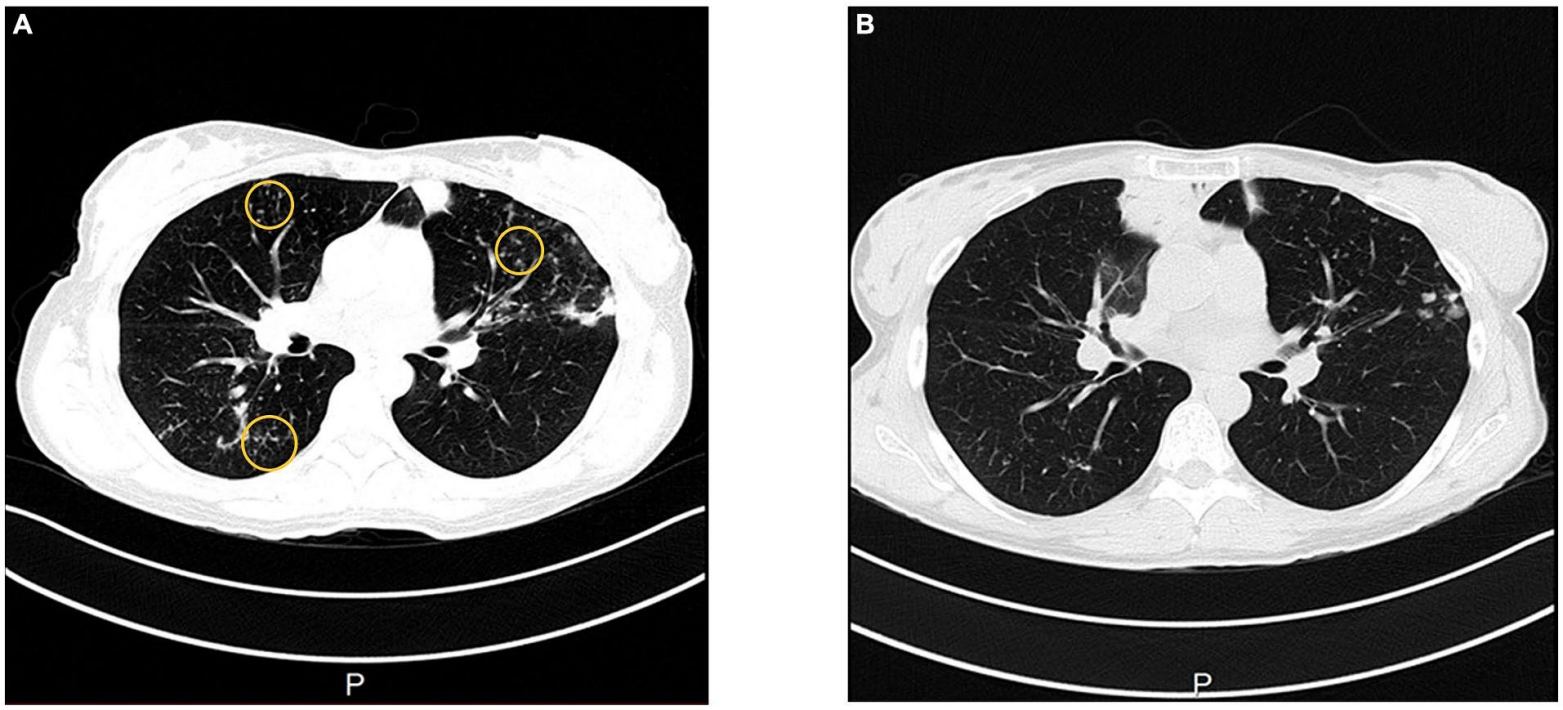
In August 2022, CT shows bronchiectasis with micronodular infiltrates with a “tree-in-bud” pattern in the right lower lobe and left lingula **(A)**, that have regressed over time in January 2023 **(B)**.

On July 27, 2022, the patient experienced a relapse characterized by fever and productive cough, NTM was confirmed again by PCR, but drug sensitivity was not retested at this point. Consequently, she was restarted on the same anti-TB medications, effectively alleviating the symptoms. An immunodeficiency workup was conducted in August 2022, and the findings are presented in Table 1. Additionally, a chest computed tomography (CT) scan was ordered to accurately evaluate the progression of her disease. The scan revealed a pulmonary cavity in the right lung (Figure 2A) and a “tree-in-bud” pattern, indicating the presence of mucus and pus causing the normally invisible peripheral airway to become visible (Figure 3A). 1 month later, a bronchoscopy with biopsy was performed to potentially identify other granulomatous diseases that resemble tuberculosis, given the patient’s recent relapse. Biopsy results showed necrotizing granuloma in the submucosa of the right upper lung. For more details, refer to Table 1.

In early January 2023, just 1 month before completing her second anti-TB course (2 months of Isoniazid, Rifampin, Pyrazinamide, and Ethambutol, followed by 4 months of Isoniazid and Rifampin), she relapsed again for a week, presenting with night sweats, fever, productive cough, and pain in the upper right chest. Subsequently, *M. riyadhense* was detected by sputum acid-fast bacilli (AFB) stain and culture, showing sensitivity to all four anti-TB medications (Table 1). Following a multidisciplinary team discussion to modify her medications, a decision was made to start Ethambutol and Pyrazinamide, along with Linezolid 600 mg and Moxifloxacin 400 mg. We also included biweekly electrocardiograms to monitor QTc for possible side effects. However, she did not opt for the new regimen, stating that she is feeling better. In addition, a consultation with a thoracic surgeon was planned to discuss the possibility of segmentectomy vs. right upper lobectomy. This consideration arises as the cavity likely functioned as a bacterial reservoir, contributing to her frequent relapses. Despite controversial radiologic findings with worsening pulmonary cavity seen in Figure 2B and regressing “tree-in-bud” pattern seen in Figure 3B, the patient appeared reluctant to undergo the latter procedure. After thorough discussion, the patient decided to wait and to get back to the hospital whenever she is ready to resume treatment.

In February 2024, the patient contacted the thoracic surgeon to proceed with right upper lung lobectomy, which was uneventful with good lung expansion and no pleural effusion. One week after discharge, she presented with fever, profuse night sweats, and productive cough, which was diagnosed as moderate right-sided pleural effusion for which she was admitted for 4 days. Ultrasound-guided thoracentesis (with pleural sampling) was performed, and the pleural fluid analysis showed lymphocytic exudate and a negative AFB stain, highly suggestive of mycobacterial pleural effusion. The previously suggested modified regimen was initiated, which includes Ethambutol 1,200 mg OD, Pyrazinamide 1,500 mg OD, Moxifloxacin 400 OD, and Linezolid 600 OD (+ Pyridoxine 10 mg OD). In March 2024, Moxifloxacin caused severe tendinitis, which was replaced by Rifampin with the rest of the regimen unchanged.

In April 2024, after the latest drug sensitivity results were received, the patient’s treatment plan was modified to Isoniazid 300 mg OD, Rifampin 600 mg OD, Ethambutol 1,200 mg OD, Pyrazinamide 1,500 mg OD, and Clarithromycin 500 mg BID. After the final treatment plan, during the subsequent follow-up visit, the patient stated that her symptoms significantly improved apart from a minimal residual cough that is slowly improving. She expressed satisfaction with the results and the plan of care.

## Discussion

*Mycobacterium riyadhense* is a nontuberculous species of bacteria belonging to the genus Mycobacterium, first identified in Riyadh, KSA, hence its name. The rising incidence of NTM infections constitutes a major epidemiological and public health threat worldwide (4). It seems that the infection is not limited to certain geographical locations since cases have been reported from France (5), Bahrain (5), and South Korea (6) in addition to Saudi Arabia.

Nontuberculous mycobacteria are generally rare, but they are a common cause of infection in immunocompromised hosts. *Mycobacterium riyadhense* has been lately described as an opportunistic infection that affects individuals with suppressed immune status as in HIV patients. Although HIV patients are prone to pulmonary infections by opportunistic pathogens in the late stage of AIDS, manifesting the disease with pulmonary infection caused by *Mycobacterium riyadhense* is extremely rare with only one case reported in the literature (7). Interestingly, two new cases have been reported in the literature with *Mycobacterium riyadhense* being the first presentation of an opportunistic infection that led to HIV diagnosis (7).

*Mycobacterium riyadhense* primarily affects the lungs but can also involve other organs. There is no evidence of human-to-human transmission yet reported (3). Not very different from other mycobacteria, symptoms of infection with *M. riyadhense* include persistent cough, fever, night sweats, asthenia, and weight loss. Diagnosis typically involves culturing the bacteria from clinical samples and performing molecular tests for accurate identification, as only culture can differentiate *M. riyadhense* from *M. tuberculosis*. Unfortunately, TB diagnosis is not always confirmed with cultures, and empirical treatment is sometimes started on a clinical basis. If this is added to reports that highlight the misidentification of NTM as *M. tuberculosis* (8), then a question arises as to whether at least some of the patients that are being treated as TB, might in fact have NTM infections. This is crucial because accurate diagnosis is essential for proper management and antibiotics choice. To date, no specific treatment regimen for *M. riyadhense* has been developed (1). Although resistance to Isoniazid is common, most patients responded well to standard anti-TB regimens and were cured (1).

In the other cases of *M. riyadhense*, incorrect diagnoses of TB were made, but in our case, misdiagnosis was prevented by PCR and culture, which excluded TB and warranted early treatment with serial laboratory and radiologic follow up. Regarding the culture medium used for the cultivation of *M. riyadhense*, a liquid medium (MGIT, “mycobacteria growth indicator tube,” in the automated MGIT 960 system, BD) was used along with two different solid media (Lowenstein-Jensen and Stone brink medium). The isolate grew on all of them. Additionally, differentiation was performed by 16S sequencing. Limited knowledge about this pathogen raises questions about its infectivity and possible drug resistance that may challenge treatment used as in our case of *M. riyadhense*, which was initially (December 2021) susceptible to all anti-TB drugs but later developed resistance. In April 2023, using sputum culture, drug sensitivity showed resistance to Ciprofloxacin, Clarithromycin, Trimethoprim-sulfamethoxazole and intermediate resistance to Doxycycline. In April 2024, using the lung tissue sample obtained during lobectomy, drug sensitivity showed resistance to Trimethoprim-sulfamethoxazole and intermediate resistance Doxycycline, Ciprofloxacin and interestingly, showed susceptibility to Clarithromycin which explains why it was added in the final regimen.

## Conclusion

Identification of this previously unknown pathogen raises concerns for human health and demonstrates the continuing threat caused by NTM. With the rising global prevalence of NTM, comes the need for accurate diagnosis and appropriate management of *Mycobacterium riyadhense* infections in the region. Therefore, clinicians should be skeptical and vigilant to the possible emergence of *M. riyadhense* as a more common pathogen.

### Patient perspective

Having experienced a complicated and rare case of Mycobacterium with three relapses over a span of more than 2 years, I found the treatment journey to be a mix of satisfaction and frustration. While the medications initially yielded a good response, the frequent relapses and the necessity of undergoing surgery left me feeling disheartened. Despite the challenges, I am immensely grateful to my doctor for their support and guidance throughout this difficult period. Currently, I am significantly improving and for that, I am truly thankful.

## Data availability statement

The datasets presented in this article are not readily available because access to the data has to be through the Institutional Review Board or the corresponding author. Requests to access the datasets should be directed to MO, Dr.michael@dmcg.edu.

## Ethics statement

The studies involving humans were approved by Institutional Research Board, Dubai Medical College, Dubai, UAE. The studies were conducted in accordance with the local legislation and institutional requirements. The participants provided their written informed consent to participate in this study. Written informed consent was obtained from the individual(s) for the publication of any potentially identifiable images or data included in this article.

## Author contributions

BS: Formal Analysis, Writing – original draft, Writing – review & editing, Conceptualization, Methodology, Project administration, Resources, Validation, Investigation. LS: Conceptualization, Formal Analysis, Investigation, Methodology, Resources, Writing – original draft, Writing – review & editing, Validation, Funding acquisition, Supervision. DA: Conceptualization, Formal Analysis, Investigation, Methodology, Resources, Validation, Writing – original draft, Writing – review & editing, Data curation, Project administration. MS: Conceptualization, Data curation, Formal Analysis, Investigation, Methodology, Resources, Writing – original draft, Writing – review & editing. SG: Formal Analysis, Investigation, Methodology, Resources, Writing – original draft, Writing – review & editing, Project administration, Supervision. SA: Conceptualization, Formal Analysis, Investigation, Methodology, Resources, Writing – original draft, Writing – review & editing, Project administration, Supervision, Funding acquisition. JG-S: Formal Analysis, Investigation, Methodology, Project administration, Resources, Supervision, Writing – original draft, Writing – review & editing, Conceptualization, Data curation, Validation. MO: Conceptualization, Formal Analysis, Methodology, Project administration, Resources, Supervision, Writing – original draft, Writing – review & editing, Validation.
